# Performance evaluation of rhamnolipids addition for the biodegradation and bioutilization of petroleum pollutants during the composting of organic wastes with waste heavy oil

**DOI:** 10.1016/j.isci.2022.104403

**Published:** 2022-05-13

**Authors:** Jianfeng Bao, Yuanfei Lv, Chenchen Liu, Shuangxi Li, Zhihong Yin, Yunjiang Yu, Liandong Zhu

**Affiliations:** 1 School of Resources & Environmental Science, Hubei International Scientific and Technological Cooperation Base of Sustainable Resource and Energy, Hubei Key Laboratory of Biomass-Resources Chemistry and Environmental Biotechnology, Wuhan University, Wuhan 430079, P.R. China; 2 State Environmental Protection Key Laboratory of Environmental Pollution Health Risk Assessment, South China Institute of Environmental Sciences, Ministry of Ecology and Environment, Guangzhou 510655, China

**Keywords:** Chemistry, Surface chemistry, Environmental chemistry

## Abstract

Environmental pollution caused by petroleum hydrocarbons is being paid more and more attention worldwide. Surfactants are able to improve the solubility of petroleum hydrocarbons, but their effects on petroleum hydrocarbon degradation in composting systems are still unclear. In this study, the effects on microbial community succession were investigated by adding petroleum hydrocarbons and rhamnolipids during composting of organic wastes. The results showed that the compost and the addition of rhamnolipids could effectively reduce the petroleum hydrocarbon content with an efficiency of 73.52%, compared to 53.81% for the treatment without addition. Network analyses and Structural Equation Model suggested that there were multiple potential petroleum degraders microbes that might be regulated by nitrogen. The findings in this study can also provide an implication for the treatment of petroleum hydrocarbon pollutants from oil-polluted soil, and the technology can be potentially applied on an industrial scale in practice.

## Highlights

• Effects of rhamnolipids on the removal of petroleum hydrocarbons were investigated
• The relationship between PDM, APDM, and environmental factors was revealed
• There was a significant correlation between nitrogen and PDM and APDM
• Rhamnolipids are bio-resources for effectively removing petroleum hydrocarbons

## Graphical abstract

**Figure:**
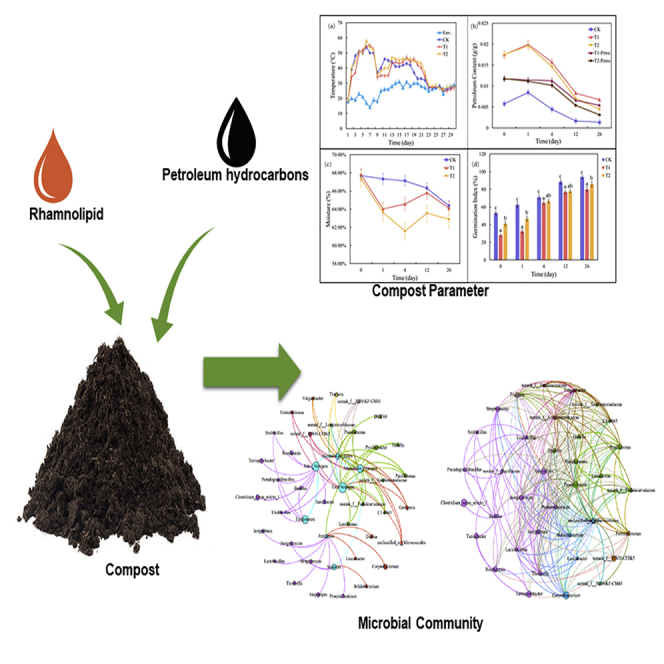

## Introduction

With the progress of industry and economy, the role of petroleum resources in the development of human society has become more and more critical (Renjith et al., 2021; Zhu et al., 2019). According to statistics, fossil fuel consumption in 2019 accounted for 80% of the world’s energy consumption, of which 33.63% was contributed by petroleum (Karakurt, 2021), and the consumption shows an upward trend (Agency and Schrder, 2019). However, in the process of oil extraction, transportation, storage, processing, and use, oil pollution problems caused by improper operation or unexpected accidents were not uncommon. Petroleum is mainly composed of alkanes, cycloalkanes, and aromatic hydrocarbons. These pollutants can usually exist in the soil for a long time and gradually be accumulated. The National Oceanic and Atmospheric Administration (NOAA) conducted a survey in 2010. In the sandy soil of the coastline of Alaska, there is still more than 87 m³ of oil, and the annual degradation rate was only 4% under natural conditions (Lim et al., 2016). When the petroleum hydrocarbon pollutants in the soil reach a certain concentration, it will inhibit the growth of microorganisms in the soil and will also form an oil film on the surface of the plant roots to hinder the plant’s absorption of nutrients (Zhang et al., 2021b; Zhu et al., 2017).

At present, there are three main methods for the remediation of petroleum hydrocarbon pollution: physical, chemical, and biological methods (Lim et al., 2016). The physical remediation method is based on adsorption to achieve the purpose of reducing the diffusion capacity of pollutants. The chemical method mainly uses strong oxidants such as hydrogen peroxide, ozone, potassium permanganate, and Fenton reagents, etc (Caniani et al., 2021; Li et al., 2021; Zhen et al., 2021). These oxidants can convert pollutants into nontoxic or low-toxic substances. However, both methods have some shortcomings, such as incomplete remediation, high cost, and risk of secondary pollution. Bioremediation technology, which is considered to be economically and environmentally friendly, has attracted more and more attention from researchers (Zhang et al., 2021a). In the process of remediation of petroleum hydrocarbons in soil, bioremediation is a commonly used technology to reduce the pollution of petroleum hydrocarbons. A previous study used soil microorganisms to degrade petroleum hydrocarbons and found that sophorolipid improved the transportation of pollutants across the microbial cell membrane, significantly increasing the bioavailability of petroleum hydrocarbons (Feng et al., 2021).

Composting is a technology applied to treat a wide diversity of organic wastes to produce fertilizer in the process of agricultural production. During the entire composting process, the microbial activity is strong, and the heat generated when the organic matter is decomposed can raise the temperature of the pile over 60 °C. Because of the thermophilic period in the composting process, which could accelerate the activity of thermophilic microorganisms, researchers have found that many organic pollutants could be degraded during the composting process. Xi et al. studied the petroleum-contaminated soil composting with biogas slurry and found that the composting process could significantly improve the degradation of total petroleum hydrocarbons in petroleum-contaminated soil, and the product is less toxic to plants (Xi et al., 2020). Some researchers also added mature compost (composting product) to the soil contaminated by petroleum hydrocarbons and found that the total petroleum hydrocarbon content of the soil decreased after adding the compost product (Yousaf et al., 2022). However, the researchers also emphasized that the use of compost alone had limited the degradation of petroleum hydrocarbons in the soil.

Although composting could promote the removal of pollutants in some cases, petroleum hydrocarbon pollutants contain multiple components that are difficult to be bioutilized. For example, linear alkanes and short-chain hydrocarbons can be utilized by a large range of microorganisms for rapid degradation (Varjani, 2017). However, some alicyclic and aromatic and long-chain hydrocarbons are difficult to be widely bioutilized (Ossai et al., 2019). Some researchers have studied the co-metabolism of pollutants by microorganisms to enhance the removal of petroleum hydrocarbon pollutants. The research results of Abed et al. showed that adding other nutrient elements in the composting process and lifting the nutritional restriction of microorganisms can effectively improve the degradation of petroleum hydrocarbon pollutants (Abed et al., 2015). Appropriately increasing the temperature of the composting system indicates that microorganisms are more active. To accelerate the degradation of petroleum hydrocarbons in the composting process, many researchers also use biochar and other additives to improve the composting performances (Duan et al., 2021; Joseph et al., 2018). Although microbial degradation of petroleum hydrocarbons can be accelerated by adding nutrient additives and increasing temperature, the low dissolution rate and hydrophobic characteristics of hydrocarbons make it difficult for microorganisms to directly contact petroleum hydrocarbons, which is the main problem to be solved. Rhamnolipid is a substance synthesized by microorganisms (*Pseudomonas aeruginosa,* etc.), which can reduce the apparent tension of the solution (Zhao et al., 2021). The hydrophilic and lipophilic groups of these surfactants can increase the hydrophobicity of bacterial cell surfaces. This makes it easier for petroleum hydrocarbons to come into contact with microbial cells. Therefore, adding rhamnolipids for degrading petroleum hydrocarbons in composting might potentially increase the contact of microorganisms with petroleum hydrocarbons, and improve the bioavailability of petroleum hydrocarbons. However, studies regarding the composting of organic wastes with waste heavy oil are still limited, and the effects of rhamnolipids addition for the biodegradation and bioutilization of petroleum pollutants are unknown in literature. Meanwhile, relative changes in the microbial community, especially degrading microorganisms during composting, are unclear as well.

Compared with the existing research, this study made a further exploration on the use of compost to degrade petroleum hydrocarbons. Based on other investigators' studies on surfactants, we designed a study using rhamnolipids to promote the degradation of petroleum hydrocarbons during composting. To explore the effect of rhamnolipid on the degradation of petroleum hydrocarbon pollutants during composting of organic wastes, this study adopted the simulated composting and set up three groups of treatments: Control (CK) without additives, Treatment 1 (T1) with petroleum hydrocarbon pollutants, and Treatment 2 (T2) with petroleum hydrocarbon pollutants and rhamnolipid. The research objectives are: (1) to evaluate the effect of rhamnolipid on the degradation of petroleum hydrocarbons; (2) to reveal the changes of microbial community during composting with rhamnolipid; (3) to explore the changes of petroleum hydrocarbon degrading microorganisms in the environment.

In this study, the C/N of compost was measured by element analyzer to evaluate the maturity in each stage. The content of petroleum hydrocarbon pollutants was analyzed by a solvent extraction and gravimetric method (Schwab et al., 1999), and the strengthening effect of rhamnolipid on the composting system was compared. Principal Component Analysis (PCA), Redundancy Analysis (RDA), Structural Equation Model (SEM), and network analysis were used to reveal the roles of the microorganisms related to petroleum degradation and the changes of microorganism communities under certain environmental factors during the composting process. This research can provide new solutions for the treatment of petroleum hydrocarbon pollution by composting of organic wastes with the addition of rhamnolipids.

## Results and discussion

### Physicochemical performances during composting

**Figure 1:**
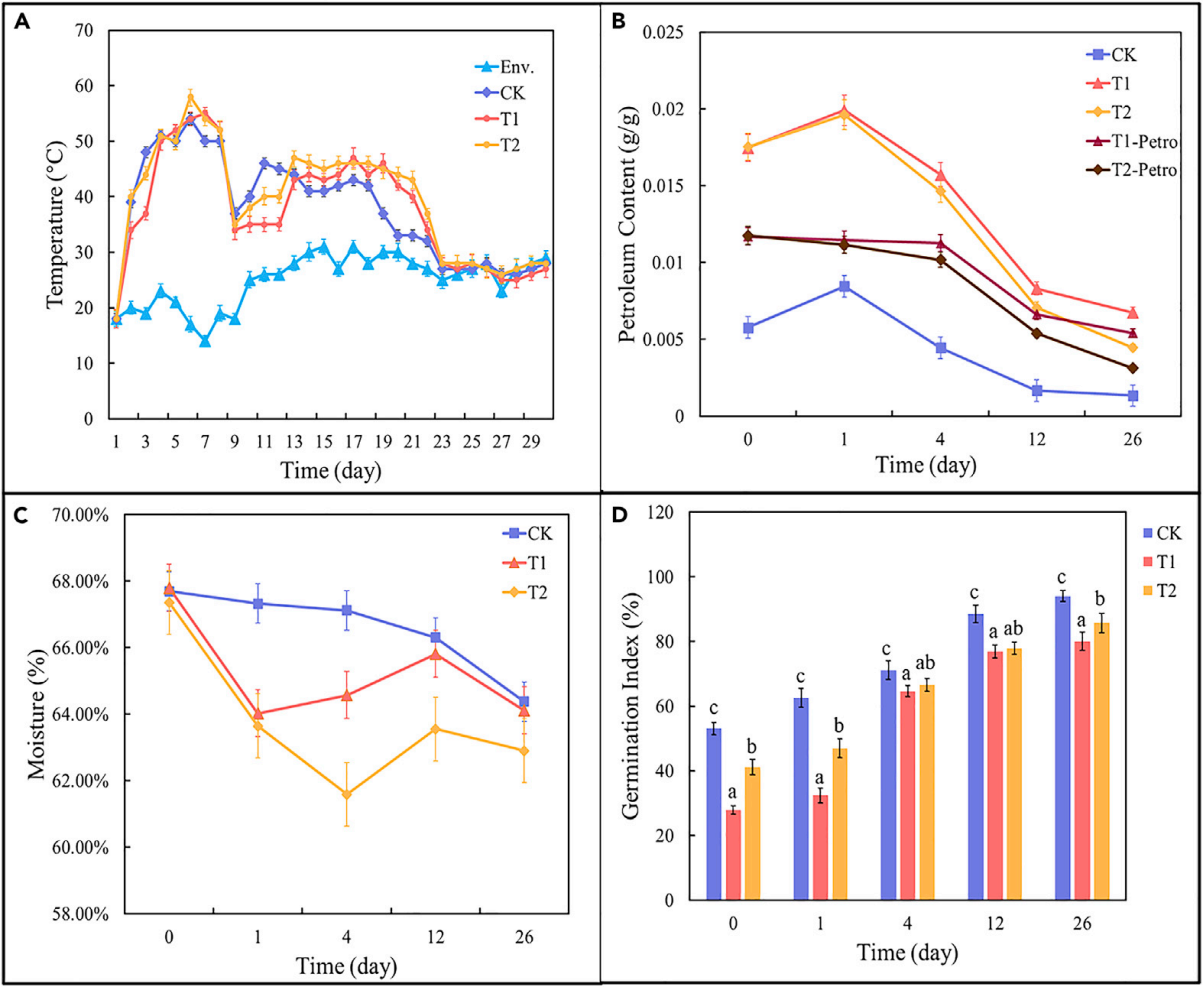
Changes of physicochemical parameters and petroleum hydrocarbon content and germination index during composting (A–D) (A) Temperature (B) Petroleum hydrocarbon content (C) Water content (D) Germination index. The error bar represents the standard deviation. Letters such as a, b and c indicate significant differences (different letters) or no significant differences (the same letters) between treatments.

The temperature obviously changed along with the composting process (Figure 1A), and the composting process could be clearly divided into four stages according to the temperature variation. The mesophilic period was from day 0 to day 1; the thermophilic period was from day 2 to day 8; the cooling period was from day 9 to day 22; and the maturity period was after day 23. Changes in temperature reflect the activity of microorganisms, and relatively high temperature means a high activity of microorganisms (Zhou et al., 2018). During the mesophilic period, the increase of temperature on CK was obviously faster than that on T1 and T2, which might be because of the addition of petroleum hydrocarbons. They might change the microbial community (Wang et al., 2022b) and inhibit the initial stage of the compost as well. There was no significant difference in thermophilic temperature among the three treatments. The highest temperature was 58°C, which appeared in the T2 group. After entering the maturity period, the temperatures of T1 and T2 were slightly higher than CK. On day 23, there was no significant difference between the temperature of each reactor and the ambient temperature, which represented the end of composting.

As shown in Figures 1B and 1A solvent extraction and gravimetric method was used for the determination of petroleum hydrocarbons. Some humic substances were also extracted when the solvent extraction and gravimetric method was used to determine the petroleum hydrocarbons in the sample. Therefore, we used T1-Petro and T2-Petro to indicate the content of petroleum hydrocarbons in T1 and T2. The content of petroleum hydrocarbons continued to decrease throughout the composting process. During the mesophilic period of the composting, microbial activity was intensified, decomposing a large amount of macromolecular organic matter into soluble substances, which could be extracted by solvents and observed in the prints of CK. The addition of rhamnolipid in T2 accelerated the decomposition of organic matter in the compost (Yin et al., 2019), so that the petroleum hydrocarbon content after composting was obviously reduced compared with T1.

During the composting process, the highest moisture content was 67.80% at the beginning of composting, whereas the lowest was 61.59% during the thermophilic period. The moisture content generally maintained between 60 and 70%, which was conducive to the normal progress of composting (Figure 1C). T1 and T2 experienced more obviously moisture loss during the mesophilic period and thermophilic phase. This was because during the thermophilic period, the microbial activity was strong and the decomposition of organic matter consumed a lot of water.

As shown in Figure 1D, the GI of the compost extract was measured. The results showed that GI gradually increased with the duration of composting, which indicated a progressive maturity of composting. According to China National Standards (GBT23486-2009), GI with more than 50% was considered as basic compost maturity in this test, whereas the 26-day GI samples with more than 50% were seen as compost maturity requirements. Among different treatments, GI accordingly reached 93.99%, 80.01, and 85.73% in CK, T1, and T2 (CK > T2> T1), which indicated the petroleum hydrocarbon germination inhibition (Haider et al., 2021). In other words, the addition of rhamnolipid could help reduce this inhibition during the composting.

### Succession of the bacterial community

**Figure 2:**
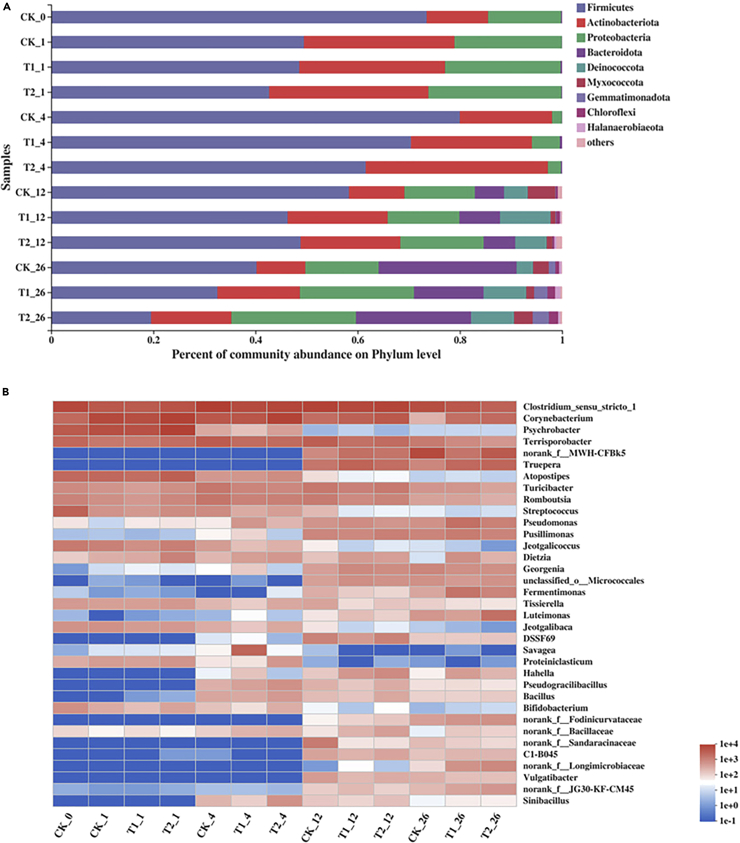
Changes in the relative abundances of bacteria during the composting (A) Phylum level. (B) Genus level during the composting.

Figure 2A shows the nine most abundant phylum level microorganisms (abundance> 1%) in all samples. Firmicutes (73.74%), Proteobacteria (14.30%), Actinobacteria (12.03%), and Bacteroidetes (0.18%) were the dominated phylum among all microorganisms in the samples. These four kinds of common phylum microorganisms represented over 99.80% of the total microbial gene sequences of bacterial 16S rRNA. Firmicutes accounted for 79.96%, 70.74, and 61.55% in CK, T1, and T2 treatments and were the most abundant bacteria in the thermophilic period. However, as the temperature decreased, the relative abundance of Firmicutes also gradually decreased. After the thermophilic period, abundance of Firmicutes decreased to 58.26%, 46.26, and 48.81% in CK, T1, and T2, respectively. A study showed that the cell wall structure of Firmicutes microorganisms could withstand the high temperature in the composting process (Zhong et al., 2017). At the same time, Firmicutes microorganisms could also decompose organic matter during the thermophilic period (Zhang et al., 2016). In this study, Firmicutes changed significantly with temperature. During the thermophilic period, Firmicutes had the highest abundance in each treatment, indicating that Firmicutes played an important role in the decomposition of organic matter during the thermophilic period. Actinobacteria is a group of microorganisms whose abundance ranked the second in each treatment. Studies showed that microbial Actinobacteria could promote the degradation of organics that were difficult to be degraded, especially for lignocellulosic materials and aromatic hydrocarbons (Bao et al., 2021).

In the study of Wei et al. (2018), it was proposed that the ratio of Firmicutes and Actinomycetes might also be considered as one of the indicators to measure the maturity of compost. In this study, the distribution of Actinomycota was significantly different in different treatments. In CK treatment, the microbial abundance of Actinomycota was less than that of T1, whereas T2 had the highest microbial abundance. This indicated that petroleum hydrocarbons might be decomposed by actinomycetes microorganisms. The addition of an active agent in T2 indicated that rhamnolipids could increase the contact between petroleum hydrocarbons and microorganisms, which might be beneficial for actinomycetes to utilize petroleum hydrocarbons. In other words, the abundance of *Actinomycota* in T2 was always higher than other treatments from the thermophilic period.

To determine the distribution of petroleum degrading microorganisms (PDM) at the genus level, the top 35 genera with relative abundance in each sample was analyzed, as shown in Figure 2B. Because of the change in temperature, composition of the microorganism genus level varies significantly. This shows that temperature change is a major factor driving the composting process microbial community succession, and this finding was also previously proposed in another study (Ge et al., 2020). Some common genera of microorganisms might be observed at the end of the compost, such as *Bacillus*, *Pseudomonas* and other microorganisms, which are considered to represent petroleum hydrocarbon degrading microorganisms (Varjani et al., 2017). At the same time, we also found that some genera had different abundances between different treatments. For example, the relative abundance of *Hahella* in CK is obviously lower than that in T1 and T2 treatments, and this genus of microorganisms could promote the decomposition of petroleum hydrocarbons in sediments (Abed et al., 2014). *Luteimonas* is a common genus in composting, which has higher abundance in T2 than CK. Studies showed that these microorganisms could usually produce surfactants, so they could promote the chain scission of long-chain alkanes (Ke et al., 2018) and also promote the degradation of other petroleum hydrocarbons (Wu et al., 2020). *Fermentmonas* is a type of facultative anaerobic microorganisms. Some researchers studied *Fermentmonas* and found that this genus of microorganisms could promote the removal of polycyclic aromatic hydrocarbons (Lu et al., 2019). *Corynebacter* was also detected in this study. This type of microorganisms could commonly use PDM. Some researchers carried out research on polycyclic aromatic hydrocarbon pollution remediation by loading *Corynebacter* onto biochar and obtained a good removal effect (Saisai et al., 2021).

### The related microbial relationship network of potential PDM

**Figure 3:**
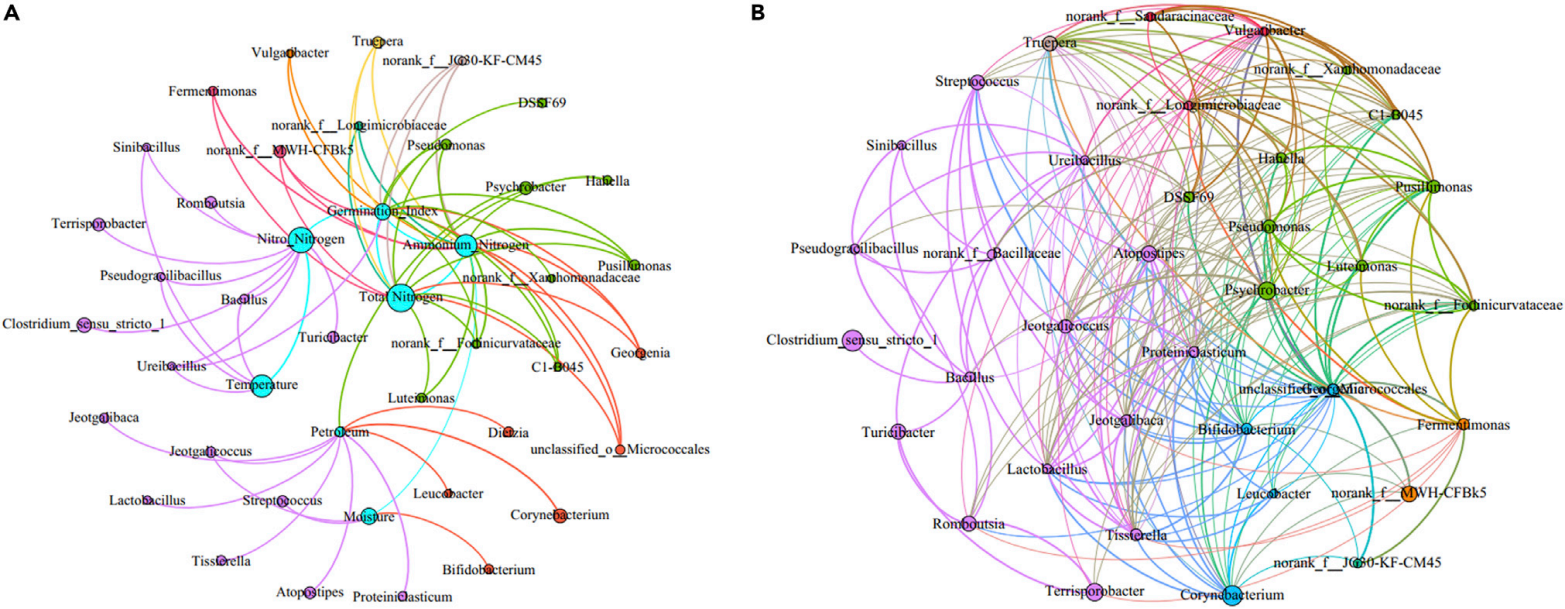
Network analysis of bacteria during the composting (A and B) (A) Network of microorganisms and environmental factors. (B) Network of microorganisms.

Network analysis studies the relationship between environmental factors and microorganisms and also explores the interconnection between dominant microorganisms. As shown in Figure 3A, there were 43 nodes and 73 edges. In Figure 3, some nodes represent environmental factors, including temperature, total nitrogen, petroleum hydrocarbon content, moisture, GI, nitrate nitrogen and ammonium nitrogen (Figures 1 and S1), the size of the nodes representing the impact of the environmental factors on microorganisms during the composting process, and another part of the nodes representing genus-level microorganisms, which are colored according to the phylum level, and the size of the nodes indicated the abundance of microorganisms. The edges in Figure 3 represent a significant positive correlation between two connected nodes, and a wider connection represents a higher correlation. It can be observed from the figure that total nitrogen had the highest influence, which was related to 13 genera, followed by petroleum hydrocarbon content, and had a correlation with 12 genera, which could be considered as potential petroleum degrading microorganisms. PDM such as *Bacillus* and *Corynebacterium* that was proven to be effective in other studies have been detected in microorganisms related to petroleum hydrocarbon content. Some researchers studied the relationship between nitrogen and petroleum hydrocarbon degrading bacteria and found that adding sufficient other nutrients (such as nitrogen) (Figure S3) could significantly promote the degradation of petroleum hydrocarbons. In this study, *Hahella* and *Bacillus*, both of which were significantly positively correlated with petroleum hydrocarbons and nitrogen content, were also found. Meanwhile, the correlation between the top 40 genera in the abundance was shown in Figure 3B. According to Pearson correlation screening, the final figure contained 37 nodes and 266 edges. A large number of connections indicated that the microbes were closely linked. It can be seen that with 12 potential PDM as the center, there were a total of 45 edges that represented a positive correlation. Among them, *Bacillus* was related to the other seven genera and was the most closely related to other microorganisms for potential petroleum degradation. Co-occurrence was observed between PDM and some other microbial genera. As to microorganisms, which belong to some other genera and are commonly related to PDM, herein we named these microorganisms associate petroleum degrading microorganisms (APDM) (Figure S4). In previous studies, it was found that some environmental factors could affect other microorganisms through indirect effects (Ting et al., 2021). It is worth mentioning that these genera closely related to *Bacillus* (including *Bacillus* itself) also had a significant positive correlation with the nitrogen content of environmental factors, which indicated that environmental factors could affect many potential PDM, indirectly affecting the degradation process of petroleum hydrocarbons.

### Relationships among bacterial community, physicochemical parameters and PDM

**Figure 4:**
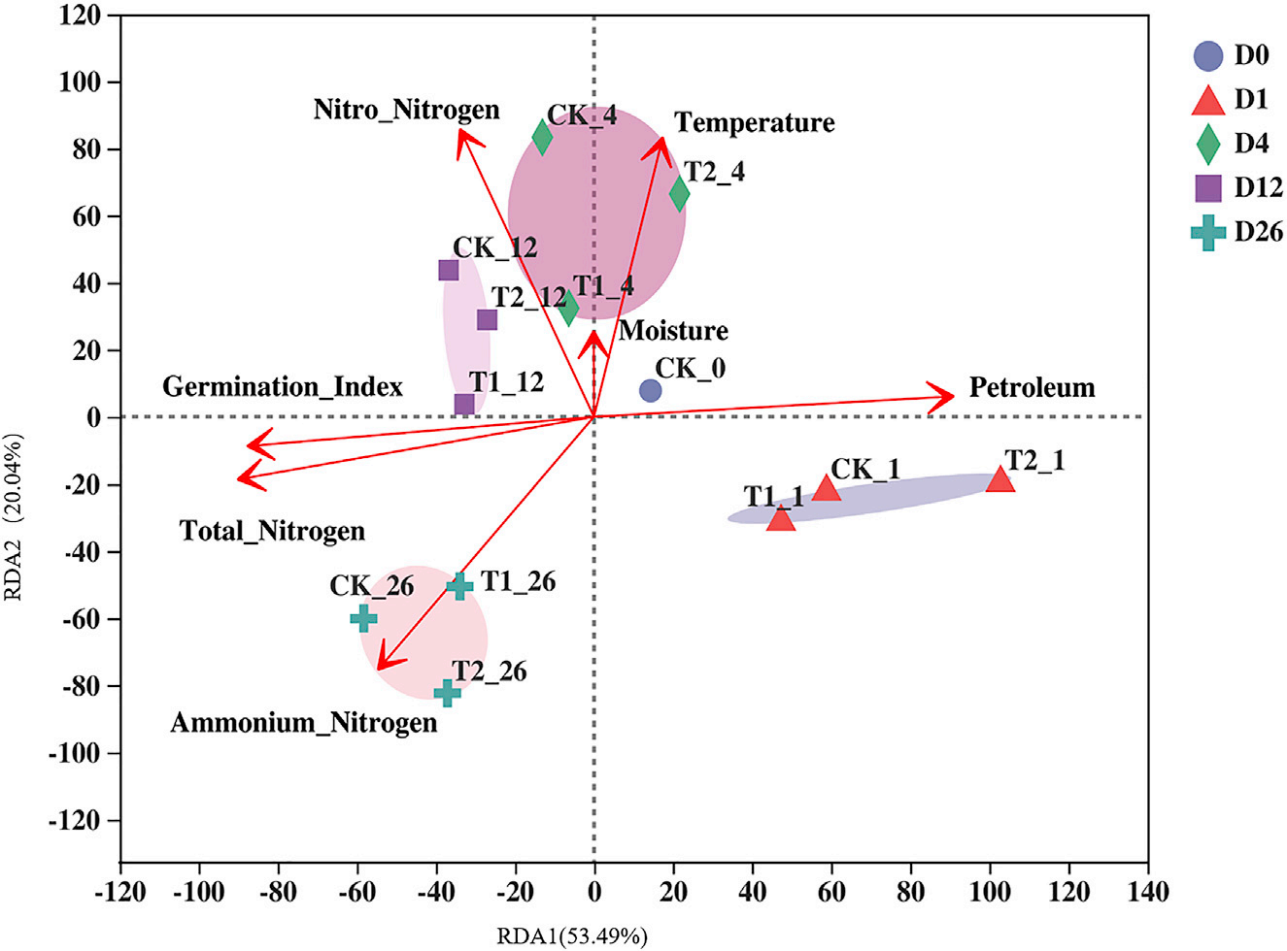
Redundancy analysis based on physicochemical parameters, microbial community, germination index, and petroleum hydrocarbon content.

In the entire composting system, there were also complex correlations between environmental factors and microorganisms (Tang et al., 2021). To explore the interaction between different factors, RDA was used to rank all the factors that might affect the microbial community and the changes in petroleum hydrocarbons. RDA1 and RDA2 explained 73.53% of the total variation (Figure 4). The samples were clustered with the composting process, indicating that the composting process was the main driving force for the succession of the microbial community (Dai et al., 2021). As the composting progressed, the petroleum hydrocarbon content gradually decreased, whereas the GI gradually increased, which confirmed the conclusion that the aforementioned compost could weaken the inhibitory effect of petroleum hydrocarbons on the GI. Figure 4 shows that the nitrogen content changed evidently during the composting process, whereas the nitrogen content had a negative correlation with the petroleum hydrocarbon content, which might mean that the nitrogen content during the composting process would affect the degradation and removal of petroleum hydrocarbons.

**Figure 5:**
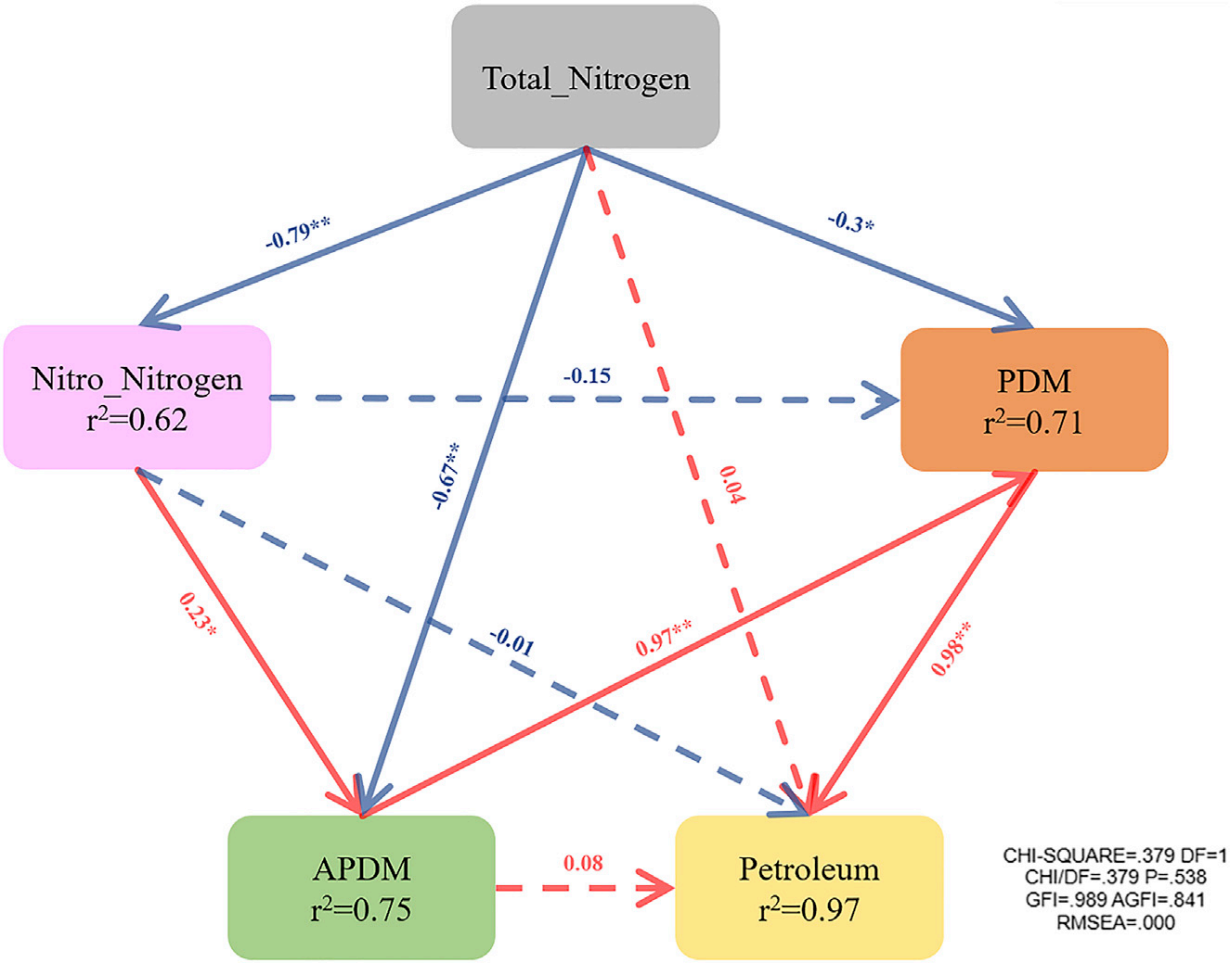
Causal relationships among physicochemical parameters, petroleum hydrocarbon content, PDM and APDM of compost process based on the SEM.

According to previous research, during composting, petroleum hydrocarbons were mainly removed because of microbial degradation (Tran et al., 2020). To explore the complex effects of petroleum degradation related microorganisms and relative environmental factors, we used total nitrogen, nitrate nitrogen, petroleum hydrocarbon content, and other indicators such as PDM and APDM to build a SEM. As shown in Figure 5, 97% of the impact of petroleum hydrocarbons could be described by the model. Among them, nitrate nitrogen had a significant positive effect on APDM, APDM on PDM, and PDM on petroleum hydrocarbons. This indicated that the increase of nutrients promoted the proliferation of APDM, and thus APDM and PDM were likely to have a symbiotic relationship. Therefore, the increase of nutrients indirectly promoted the degradation of petroleum hydrocarbons. This model results also confirmed the conclusion drawn in Figure 5. Meanwhile, we also found that some negative effects existed between total nitrogen and nitrate nitrogen, between total nitrogen and APDM, and between total nitrogen and PDM. This did not mean that when the total nitrogen was high, it had an inhibitory effect on these factors because of nitrogen conversion and the absorption and utilization of nitrogen by microorganisms.

### Limitations of the study

This study innovatively applied the surfactant rhamnolipid to enhance the degradation of petroleum pollutants in composting systems. In this study, 16S rDNA sequencing technique was applied to analyze the oil-degrading microbiota during the composting process, whereas metagenomics method was not used, presenting the main limitation of this study. The functions and metabolic pathways of different microorganisms during the degradation of petroleum hydrocarbons can be explored by metagenomics. In the future, metagenomics can also reveal the respective roles of different microbial populations as well as the synergistic relationships between populations during the degradation of petroleum hydrocarbons. This study reveals the relationship between petroleum hydrocarbons and nutrients during the composting process, providing a theoretical basis for the exploration of more efficient control of petroleum hydrocarbon pollution in future by using surfactants to accelerate the degradation of petroleum hydrocarbons.

## Conclusion

The addition of rhamnolipids could promote microorganisms’ degradation of petroleum hydrocarbons and reduce the toxicity of composting products to plants. In terms of the composition of the microbial community, the addition of rhamnolipids could significantly increase the abundance of PDM and enhance the connection between PDM and APDM. The content of easily available nitrogen sources such as total nitrogen and nitrate nitrogen indirectly affected the content of total petroleum hydrocarbons by affecting potential PDM and related microorganisms. The finding indicated that the addition of rhamnolipid could promote the degradation of petroleum hydrocarbon pollutants by microorganisms, and rhamnolipid addition was an effective method in repairing petroleum hydrocarbons.

## STAR★Methods

### Key resources table

**Table:** 

REAGENT or RESOURCE	SOURCE	IDENTIFIER
**Biological samples**		

Compost samples	This paper	N/A

**Chemicals, peptides, and recombinant proteins**		

rhamnolipid	Meryer Chemical Technology	CAS:869062-42-0

**Critical commercial assays**		

E.Z.N.A.® soil DNA Kit	Omega Bio-tek	D5625-00

**Deposited data**		

Raw and analyzed data	This paper	N/A

**Oligonucleotides**		

primer pairs 338F (5'-ACTCCTACGGGAGGCAGCAG-3') and 806R(5'-GGACTACHVGGGTWTCTAAT-3')	Majorbio	https://cloud.majorbio.com/

**Software and algorithms**		

fastp	(Pearson, William R., 1990)	https://github.com/Open/fastp
FLASH	(Xie J et al., 2021)	http://www.cbcb.umd.edu/software/flash
R3.1.0.	(Ting et al., 2021)	https://www.r-project.org/
Origin 2021	(Wang G et al., 2022a)	https://www.originlab.com/
IBM SPSS Amos	(Zhao, W et al., 2022)	https://www.ibm.com/cn-zh/products/spss-statistics
Gephi 0.9.2.	(Wang G et al., 2022a)	https://gephi.org/

**Other**		

Sequence data, analyses	This paper	Accession Number: SRP372998

### Resource availability

#### Lead contact

Further information and requests for resources should be directed to and will be fulfilled by the lead contact, Jianfeng Bao: jianfeng_bao@foxmail.com.

#### Materials availability

This study did not generate new unique reagents.

### Method details

#### Reactor operation

Raw materials for composting include swine manure, wheat straw, waste heavy oil and rhamnolipids. The swine manure comes from a farm near Shijiazhuang City, Hebei Province, China, while the waste heavy oil comes from an oil refinery near Jingmen City, Hubei Province, China. Before the start of the experiment, the chemical properties of swine manure and wheat straw were determined. The experiment was conducted in a greenhouse of the Wuhan University, China. Three groups of composting boxes with a size of 40 × 50 × 48 cm were used to simulate composting. To ensure oxygen supply, there were 4 ventilation holes of 2 cm∗2 cm on the six sides of the reactor. Meanwhile, the whole compost was manually ventilated every 8 h.

The reactor was equipped with an insulating layer to ensure compost process normally. Laboratory scale compost simulation is difficult to be carried out without external heating or insulation, since the temperature will quickly dissipate due to lack of insulation. The moisture of the reactor was adjusted to 60% and the C/N was 30:1. Three treatments were set in the test. No waste heavy oil and rhamnolipid were added into CK, and 2%wt heavy oil was added into T1, while 2%wt heavy oil and 5%wt rhamnolipid were added into T2. The temperature at the center of each reactor was measured every day, and the environmental temperature was also measured at the same time. The stage of compost shall be judged, according to the temperature, and water supplemented to keep the moisture content of the pile at 60%.

#### Sample collection and storage

Three replicates were taken and 500 g sample was collected on Day 0, 1, 4, 12 and 26, respectively. After collection, all samples were divided into two parts and stored at 4°C and −20°C for determination of physical and chemical properties and extraction of DNA. All samples were freeze-dried in a frozen dryer and crushed by an ultra-centrifugal mill (ZM200, Retsch, Germany) to ensure that the particle diameter was not greater than 1 mm before DNA extraction.

### Quantification and statistical analysis

#### Physicochemical parameters

In addition to the reactor temperature, the content of petroleum hydrocarbon pollutants was analyzed by the solvent extraction and gravimetrical method. The moisture content was determined by weighing the weight loss after drying at 105°C for 8 h to make the sample reach a constant weight. The total nitrogen content was determined by the Kjeldahl method. The total carbon content was determined by an elemental analyzer.

In this study, the petroleum hydrocarbon content in the sample is explored based on the extraction method, but the substances that can be extracted by the solvent in the compost are not only petroleum hydrocarbon pollutants, T1 represents all the extracted organic matter content, and T1-petro represents the extracted petroleum hydrocarbon pollutants content in the sample, calculated as follows:

$$T1-\text{petro }=\;\frac{\sum_{\text{i}=1}^{\text{n}}T1-\sum_{\text{i}=1}^{\text{n}}CK}{\text{n}}\left(\text{n}=3\right)$$

$$T2-\text{petro }=\;\frac{\sum_{\text{i}=1}^{\text{n}}T2-\sum_{\text{i}=1}^{\text{n}}CK}{\text{n}}\left(\text{n}=3\right)$$

n：Number of repeats of the treatment.

#### Germination index

The 20 g compost samples were added into 200 mL of distilled water, shaken for 20 min and dipped for 30°C for 12 h, and isolated supernatant was used for centrifugation. Eqularge filter paper was placed in the plate, evenly covered with 20 healthy seeds. And 5 mL of the above extract was taken into the plate (distilled water as a control experiment), and each treatment was repeated for 3 times.

#### High throughput sequencing analysis

The microbial community uses the 16S rDNA sequencing method to extract DNA to analyze the V3-V4 region of the sample. Primer information and test details refer to our previous research (Bao et al., 2020). Microbial community genomic DNA was extracted from compost samples using the E.Z.N.A.® soil DNA Kit (Omega Bio-tek, Norcross, GA, U.S.), according to manufacturer’s instructions. The DNA extract was checked on 1% agarose gel, and DNA concentration and purity were determined with NanoDrop 2000 UV-vis spectrophotometer (Thermo Scientific, Wilmington, USA). The hypervariable region V3-V4 of the bacterial 16S rRNA gene were amplified with primer pairs 338F (5'-ACTCCTACGGGAGGCAGCAG-3') and 806R(5'-GGACTACHVGGGTWTCTAAT-3') by an ABI GeneAmp® 9700 PCR thermocycler (ABI, CA, USA). The PCR amplification of 16S rRNA gene was performed as follows: initial denaturation at 95°C for 3 min, followed by 27 cycles of denaturing at 95°C for 30 s, annealing at 55°C for 30 s and extension at 72°C for 45 s, and single extension at 72°C for 10 min, and end at 4°C. The PCR mixtures contain 5 × Trans-Start FastPfu buffer 4 μL, 2.5 mM dNTPs 2 μL, forward primer (5 μM) 0.8 μL, reverse primer (5 μM) 0.8 μL, Trans-Start FastPfu DNA Polymerase 0.4 μL, template DNA 10 ng, and finally ddH2O up to 20 μL. PCR reactions were performed in triplicate. The PCR product was extracted from 2% agarose gel and purified using the AxyPrep DNA Gel Extraction Kit (Axygen Biosciences, Union City, CA, USA), according to manufacturer’s instructions, and quantified using Quantus™ Fluorometer (Promega, USA).

The raw 16S rRNA gene sequencing reads were demultiplexed, quality-filtered by fastp version 0.20.0 and merged by FLASH version 1.2.7 with the following criteria: (i) the 300 bp reads were truncated at any site receiving an average quality score of <20 over a 50 bp sliding window, and the truncated reads shorter than 50 bp were discarded, reads containing ambiguous characters were also discarded; (ii) only overlapping sequences longer than 10 bp were assembled according to their overlapped sequence. The maximum mismatch ratio of overlap region is 0.2. Reads that could not be assembled were discarded; (iii) Samples were distinguished according to the barcode and primers, and the sequence direction was adjusted, exact barcode matching, 2 nucleotide mismatches in primer matching.

Operational taxonomic units (OTUs) with 97% similarity cut off [3, 4] were clustered using UPARSE version 7.1, and chimeric sequences were identified and removed. The taxonomy of each OTU representative sequence was analyzed by RDP Classifier version 2.2 against the 16S rRNA database (eg. Silva v138) using confidence threshold of 0.7.

#### Data and statistical analyses

The Spearman's correlation between microbial data and environmental factors was analyzed, while screened data with significant correlation (p < 0.05) and performed data dimensionality reduction by IBM SPSS 20.0 were performed. Principal Component Analysis (PCA) and heatmap analysis were conducted with R3.1.0. while PCA was used to describe the relationship between microbes and heatmap was used to express the relative abundance of microbial phylum levels and genus levels. Redundancy Analysis (RDA), performed by Origin (2021) which could show the relationship between microorganisms and various environmental factors. Network analyses, performed by Gephi 0.9.2. which based on the Spearman’s correlation coefficients (p < 0.01) between environmental factors and genera with relative abundances >1%. The Structural Equation Model SEM was established using IBM SPSS Amos (Version 24).

## Data Availability

• The sequencing data were deposited into the NCBI Sequence Read Archive database Accession numbers are listed in the key resources table. • This study did not generate new code. • Any additional information required to reanalyze the data reported in this paper is available from the lead contact upon request.
