# Supplementary material for: Performance evaluation of rhamnolipids addition for the biodegradation and bioutilization of petroleum pollutants during the composting of organic wastes with waste heavy oil
Source: iScience. 2022 May 13;25(6):104403. doi: 10.1016/j.isci.2022.104403 (PMC9157225; doi:10.1016/j.isci.2022.104403)
Supplement: Document S1. Figures S1–S5 [file mmc1.pdf]

iScience, Volume *25*

Supplemental information

Performance evaluation of rhamnolipids addition for the biodegradation and bioutilization of petroleum pollutants during the composting of organic wastes with waste heavy oil

Jianfeng Bao, Yuanfei Lv, Chenchen Liu, Shuangxi Li, Zhihong Yin, Yunjiang Yu, and Liandong Zhu

Compost strengthens petroleum pollutant degradation: Rhamnolipids improves the bioutilization of waste heavy oil

Jianfeng Bao[a], Yuanfei Lv[a], Chenchen Liu[a], Shuangxi Li[a], Zhihong Yin[a], Yunjiang Yu[b], Liandong Zhu[a, *]

[a] School of Resources & Environmental Science, Hubei International Scientific and Technological Cooperation Base of Sustainable Resource and Energy, Hubei Key Laboratory of Biomass-Resources Chemistry and Environmental Biotechnology, Wuhan University, Wuhan, 430079, P.R. China

[b] State Environmental Protection Key Laboratory of Environmental Pollution Health Risk Assessment, South China Institute of Environmental Sciences, Ministry of Ecology and Environment, Guangzhou, 510655, China

Emails: jianfeng_bao@foxmail.com

[*] Corresponding author: ldzhu@whu.edu.cn

Fig. S1

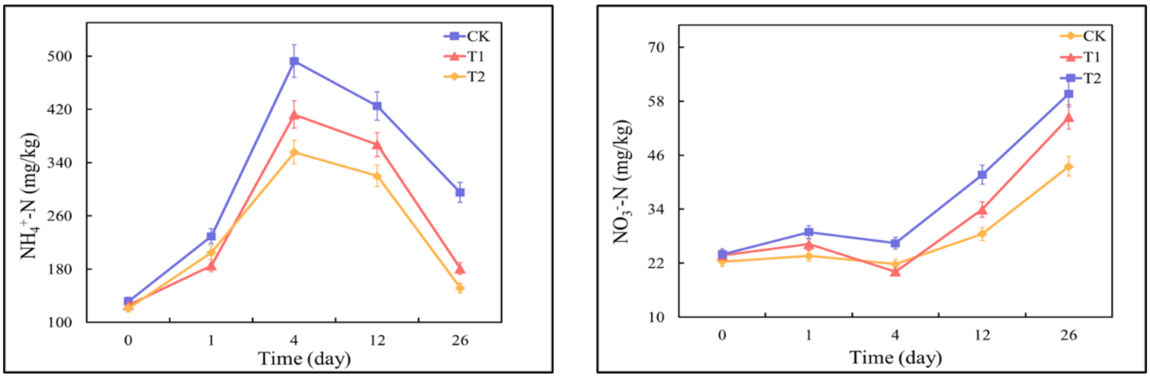

Changes of ammonium and nitrate during composting. (Fig.S1 changes in ammonium and nitrate content correlated with Fig.4)

Fig. S2

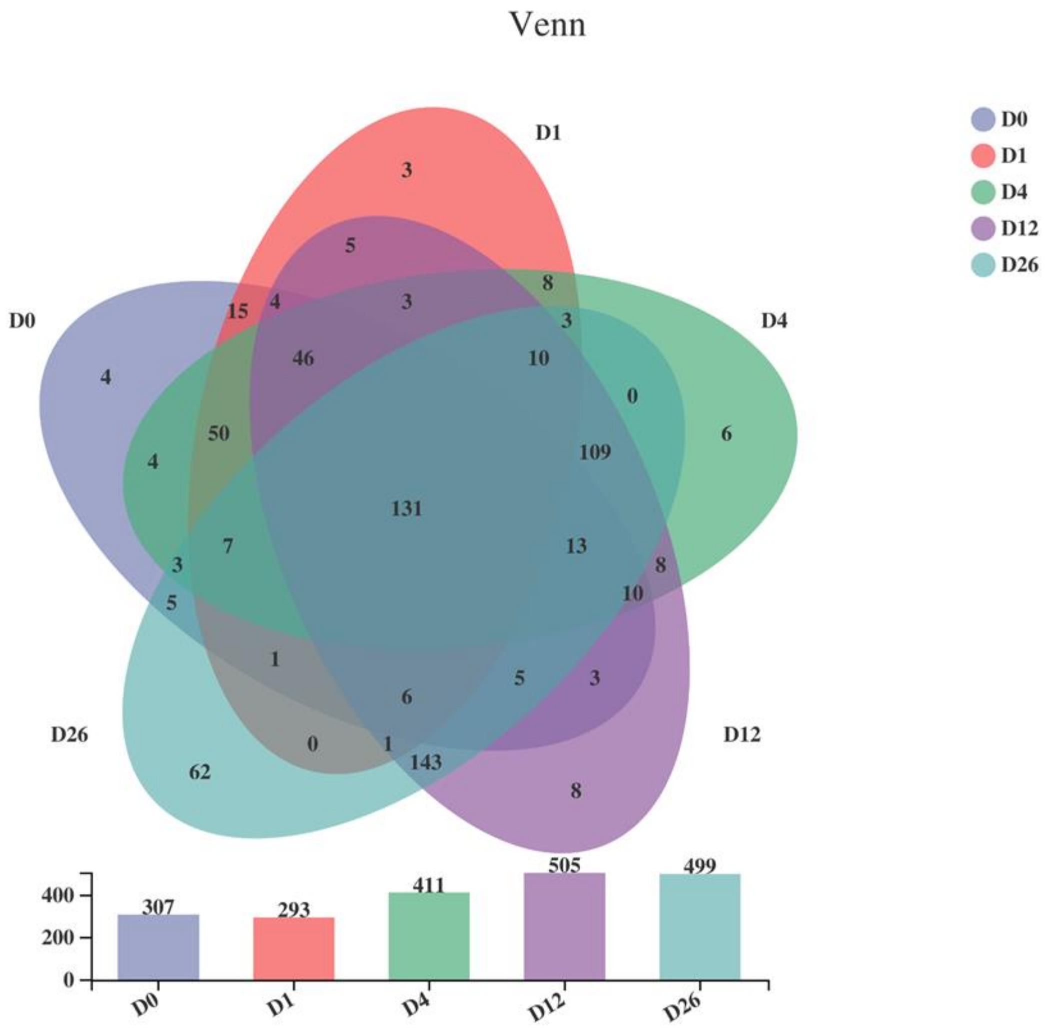

Venn diagram of microbial communities in different sampling days. (The number of microbial genera in different samples in Fig.S2 was associated with Fig.4)

Fig. S3

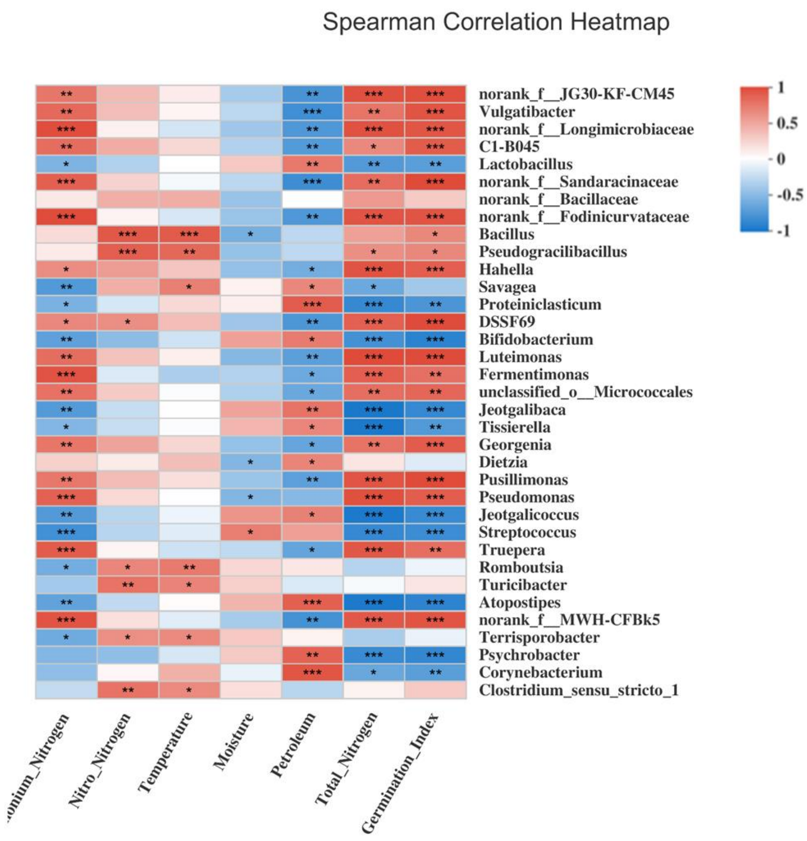

Spearman correlation heat map of various physicochemical parameters and microbial genera. (Correlations between Fig.S3 microorganisms and physicochemical parameters were correlated with Fig.3a)

Fig. S4

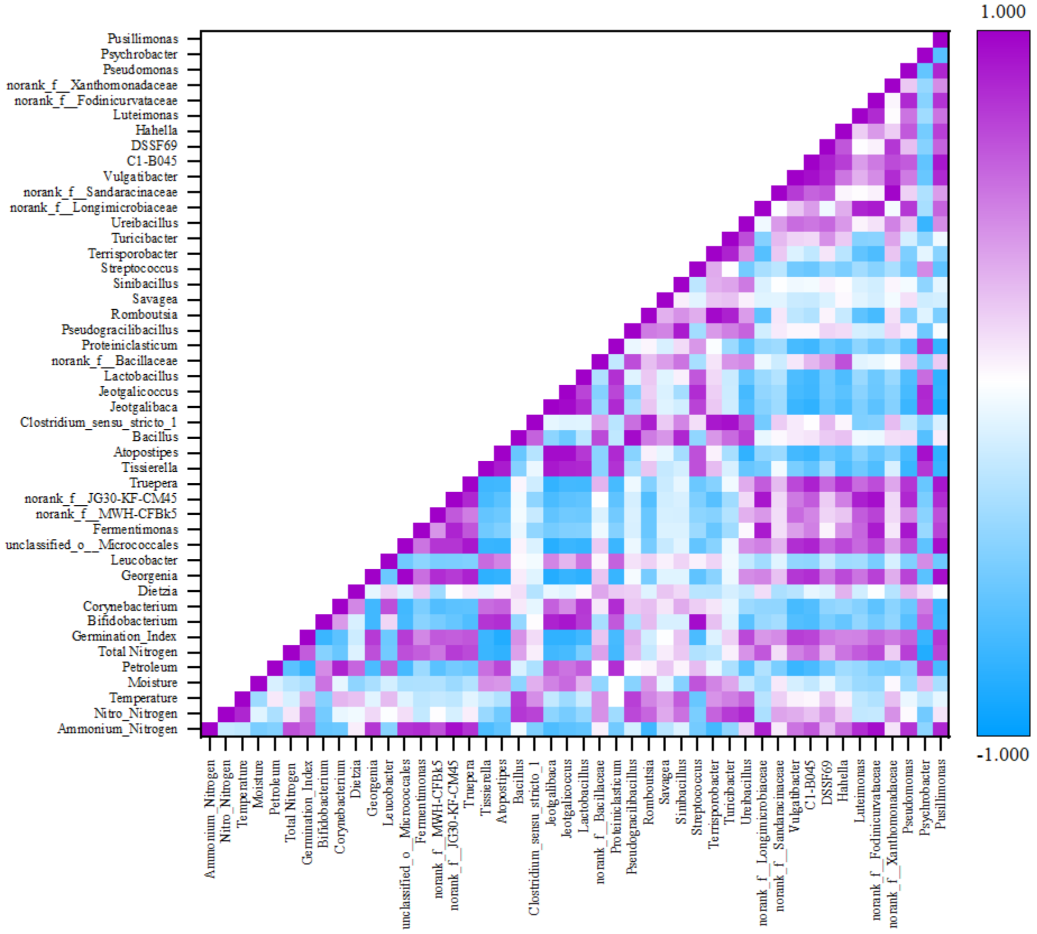

Spearman correlation heat map among microorganisms. (Fig.S4 genus-level correlations between microorganisms were associated with Fig.3b)

Fig. S5

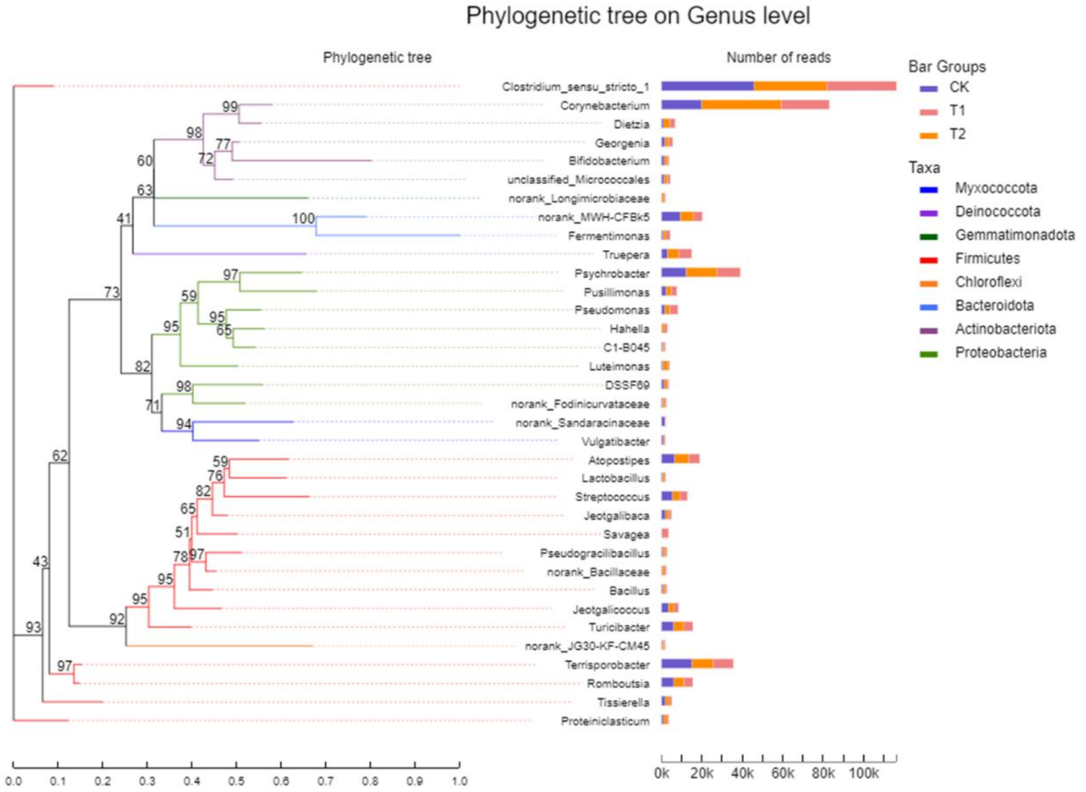

Phylogenetic tree at the level of microbial genus distribution according to treatment.

(The Fig.S5 microbial genus-level phylogenetic tree was associated with Figure Fig.2)
